# Sustainable Smart Irrigation System (SIS) using solar PV with rainwater harvesting technique for indoor plants

**DOI:** 10.1371/journal.pone.0316911

**Published:** 2025-03-21

**Authors:** Syed Zahurul Islam, Muhammad Saufi Bin Kamarudin, Mohd Noor Bin Abdullah, Mimi Mohaffyza, Lai Chee Sern, Mohammad Lutfi Othman, Jasim Uddin

**Affiliations:** 1 Faculty of Electrical and Electronic Engineering, Universiti Tun Hussein Onn Malaysia, Parit Raja, Johor, Malaysia; 2 Faculty of Technical and Vocational Education, Universiti Tun Hussein Onn Malaysia, Parit Raja, Johor, Malaysia; 3 Advanced Lightning, Power and Energy Research (ALPER), Faculty of Engineering, Universiti Putra Malaysia, Selangor, Malaysia; 4 EM-RFMic Engineering Group, Cardiff School of Technologies, Cardiff Metropolitan University, Cardiff, United Kingdom; Agricultural Sciences and Natural Resources University of Khuzestan, IRAN, ISLAMIC REPUBLIC OF

## Abstract

The project aims to develop a sustainable smart irrigation system (SIS) for the indoor plant irrigation by integrating photovoltaic (PV), internet of things (IoT), and rainwater harvesting techniques. The addressed problem involves the inconsistency and tediousness of manual watering, emphasizing the need for a sustainable design for a SIS. The IoT system consists of soil moisture sensor with GSM module powered by PV and an algorithm was developed to adjust irrigation schedules based on soil moisture data. The objectives of this project are to design and optimize the PV-powered irrigation system and implement an Arduino-enabled automatic system with SMS-triggered functionality. The methodology involves system modelling for water requirements and sizing of PV, battery, pump, and MPPT based on the load demand. The rainwater harvesting structure designed ensures water sustainability for plants’ irrigation. The system is then implemented using moisture and ultrasonic sensors managed by Arduino Uno embedded system. The electrical performance of the PV was analyzed on both cloudy and moderately luminous days, with irradiance ranging from 250.4 to 667.8 and 285.5 to 928 W/m^2^, respectively. The average output voltage and current of the battery were observed to be 13.04 V and 0.37 A (cloudy), and 13.45 V and 0.47 A (moderate) days, respectively. The rainwater collection test revealed more than 36 L in the tank after one week, indicating it could sustain watering the three plants for 72 days. Based on the analysis, the project can save 14.97 kgCO_2_ emissions per year compared to the current emissions released into the environment. The overall cost of the system is approximately RM670 (US$139.50). The SIS aligns with SDG 7, promoting affordable and integrates with 12^th^ Malaysia Plan for more efficient and environmentally friendly agricultural and water management practices.

## Introduction

In recent years, there has been an increasing interest in smart watering solutions for indoor plants. The water requirements of plants are monitored and controlled by these systems, guaranteeing optimal development and minimizing water waste [1–3]. It is critical to create effective and sustainable strategies for managing water resources in these contexts as demand for indoor gardening and urban farming grows [4,5]. Smart irrigation system (SIS) offers various benefits such as enhanced air quality and visual appeal. It relies on advanced technologies like sensors and timers to ensure precise and efficient watering. By utilizing soil moisture sensors, for example, real-time monitoring and adjustment of irrigation schedules become possible, effectively preventing both under and overwatering [6]. Moreover, research has explored the integration of smart technologies, such as IoT enabled smart irrigation controllers equipped with weather-based algorithms and wireless connectivity [7]. This integration enables adaptive watering based on environmental conditions, optimizing water usage, and minimizing plant stress. Additionally, advancements in micro-irrigation systems, like capillary wick systems, have been investigated as sustainable methods for consistently delivering water to plants and maintaining optimal moisture levels. Overall, indoor plant irrigation systems have significantly evolved by incorporating automated technologies and smart features to promote plant health. The integration of soil moisture sensors, smart controllers, and capillary wick systems has proven effective in maintaining optimal moisture levels, but further research is needed to understand their long-term impact on plant growth and sustainability [8].

A number of cross-sectional studies prove an association between agriculture and smart devices, based on the observation in literature over the past two decades. Table 1 provides a synopsis of previous studies on SIS, including the research location, methods, and outcomes of each respective study.

**Table 1:** Summary of previous research in smart farming. Sources are from 2019–2024.

Year	Ref.	Location	Method	Significant outcomes
2019	[8]	Xiaotangshan, China	∙ Water saving technology ∙ Wireless sensor enabled drip irrigation ∙ Tomato production	∙ Higher yields ∙ Less water irrigation ∙ Tomato production with andwithout smart system was 41.23 and31.58 kg/m^3^, respectively
2019	[14]	Cebu, Philippines	∙ Smart irrigation using sensors, Arduino, IoT, and mobile ∙ Robusta coffee plantation ∙ 2 months experiment	∙ SIS improves 72.56% in plant heightand number of leaves ∙ Improved compared to manualmethod (65.29%)
2021	[4]	Germany	∙ Design of a SIS for 5000 of young trees	∙ Projected to reduce waterrequirements by 1 million liters
2021	[6]	Kolkata, India	∙ Solar-powered water pump with micro-controller	∙ Integrates solar-powered water pumpwith automatic water flow control ∙ Enhancing irrigation precision basedon soil moisture levels
2021	[9]	Thessaloniki, Greece	∙ Intelligent irrigation system in agriculture 4.0 ∙ Implemented in AREThOU5A IoT platform	∙ IoT with machine learning is deployedin water management ∙ Coalesce of 5G and RF EH technology
2022	[5]	India	∙ Intelligent automatic irrigation system ∙ Soil moisture, humidity, temperature, and rainfall sensors are used ∙ Motor pump is controlled using mobile application	∙ Soil moisture, rain, temperature, andhumidity were measured ∙ Monitored through mobile apps. ∙ Web interface is developed inThinkSpeak cloud platform
2023	[2]	Brassica Juncea, Vietnam	∙ Smart greenhouse with IoT ∙ Mobile application	∙ Automatic fertilization ∙ Plant watering management
2023	[3]	Sri Lanka	∙ Develop a low-cost IoT component ∙ Wifi enabled sensor node is developed using light intensity, temperature, and humidity sensor	∙ Greenhouse environment controlapplication ∙ Control 3 − *ϕ* AC motor blower usingIoT
2023	[21]	Majmaah, Saudi Arabia	∙ Smart animal monitoring service ∙ RFID, GPS, and sensor technology ∙ Zoos and aquariums	∙ Temperature and humidity rate of zooenvironment are analyzed ∙ Heartbeat rate and other relatedparameters are measured formonitoring health of cattle
2023	[22]	Indore, India	∙ Robotic and automatic system for hydroponic farming ∙ Sensors used - pH, ultrasonic, temperature, electrical conductivity, and water flow ∙ Vertical hydroponic farming	∙ IoT assists farmers by continuouslyidentifying water levels and othercontaminants ∙ Data is monitored via a centralizedserver using the Android mobile app.
2024	[20]	Cyberjaya, Malaysia	∙ LoRa-enabled IoT solutions for smart farms ∙ Network is formed using Arduino Uno-LoRa and ESP32-LoRa device and then transferred data to ThinkSpeak cloud ∙ Cloud data is accessed through Blynk mobile apps	∙ Considering frequency, distance, andenvironment, LoRa showsexcellent performance in fruit andvegetables farming ∙ LoRA shows as cost-effective IoTsolution for farmers
2024	[23]	Tamale, Ghana	∙ Analyzing the factors driving greenhouse technology ∙ Varied impacts on household welfare	∙ Improving the adoption of GHT canenhance household welfare in Ghana
2024	[25]	Middle Atlas, Morocco	∙ Cloud based SIS - linking distributed farms using big data ∙ IoT, embedded systems, WSN node - fire, PIR, soil moisture, temperature, and humidity sensors ∙ Solar PV with battery for powering SIS	∙ Rain forecast from humidity data,which used for minimizing waterusage through smart irrigation ∙ Water level in basin is automated ∙ Sustainable energy system forenvironmental impact reduction
2024	[26]	IIT Mandi, India	∙ Soil moisture data retrieve with weather forecasting ∙ Smart irrigation schedule with cloud system	∙ Water and energy saving up to 9.24% ∙ Energy management is improvedusing solar PV
2024	[32]	Punjab, India	∙ Smart technology- digital twins ∙ IoT with digital twins is applied in smart irrigation	∙ Significant improvement in soil,weather, and crops ∙ Improve in farm operation and reducewater consumption

There is a growing body of literature that recognises on delivering power to the IoT sensor nodes deployed on the outdoor environment using radiofrequency energy harvesting technique [9]. By drawing on the concept of IoT, Achilles D. et al. have been able to show that AREThOU5A can be used for this technique to perform the intelligent irrigation system. Others performed a similar series of experiments between 2017-2020, such as IoT agnostic ecosystem [10], precision algorithm in agriculture platform using open source protocol [11] and dynamic model for water management in agriculture [12]. Researchers also proposed prediction algorithm on rainfall pattern and climate change using AI and 6G in smart irrigation [13]. Sitharthan R et al. recent analysis of 6G and AI provide a strong critique of an IoT framework, which is developed using PHP language with MySQL database managed by Apache web. The comprehensive study found that the server can store climate estimating information like temperature, mugginess, shadiness, and UV Index. The system has achieved an accuracy, sensitivity, and precision of 86.34, 89.2, and 91%, respectively. In 2023, a seminal article was published entitled coffee seedlings *(Coffea canephora)* plant parameters in Philippines and improved the coffee growth using SIS [14]. The comprehensive results by Alden Q. Gabuya et al indicate that almost 19% of water usage reduction after using the SIS with an improved height and growth rate of 72.56% and 65.29%, respectively. The smart system is implemented using wireless sensor network (WSN) to monitor soil moisture, ambient temperature, and humidity over the two months. For IoT, ThinkSpeak application software is used from importing data from remote field to cloud. In another research, a smart drip irrigation system is proposed using IoT and web-based application for monitoring and water usage control in the application of agriculture, parks, and lawns [15]. The system is designed for two plants using two moisture sensors, whose data are imported to the cloud using Wifi communication. The research also investigated linear regression between sensor and soil dry condition. Based on the sensor data, a submergible pump is controlled for watering the plants. Siddagangaiah has made efforts to develop a plant health monitoring system employing IoT technology [16]. This system enables the tracking of atmospheric conditions such as light intensity, temperature, humidity, etc., via an IoT cloud platform, with subsequent notifications sent to mobile devices.

The significance of a newly created dataset focused on rice pests was analyzed by Quach LD et al., which aids in the development of smart farming systems [17]. This dataset includes information on various pests affecting rice crops, such as insects and diseases, along with their characteristics and distribution. By utilizing this dataset, researchers and farmers can better understand pest dynamics, enhance pest management strategies, and ultimately improve rice crop yields. The integration of such data into smart farming systems enables real-time monitoring and targeted interventions, leading to more sustainable and efficient agricultural practices. Overall, this dataset serves as a valuable resource for advancing precision agriculture and addressing challenges in rice cultivation. Kenny Kueh Yung Shin et al. provide in-depth analysis of a SMART GROW system showing its relevance to an affordable automated hydroponic setup designed for urban farming in Malaysia [18]. In their experimental analysis, the system was demonstrated significant improvements in crop yield and growth rate compared to traditional soil-based methods. Its low-cost design and automated features-pH, EC, and water level sensors with ESP32 microcontroller make it accessible to urban farmers, offering the potential to increase food production in limited spaces while reducing water usage and environmental impact. The results highlight the potential of SMART GROW to revolutionize urban agriculture for cultivating fresh produce locally. The development of an expert system for the diagnosis of sugarcane diseases *(eg. Eyespot, Leaf Scald, Yellow Leaf, and Pokkah Boeng)* using machine learning techniques was proposed in India by Athiraja Atheeswaran et al. [19]. Through image processing and ANN, the system achieved high accuracy rates in identifying various sugarcane diseases based on symptoms and other factors. By leveraging machine learning algorithms, the expert system demonstrated its efficacy in aiding farmers, enabling prompt action to mitigate disease outbreaks and optimize crop management practices. These results highlight the potential of machine learning-driven expert systems to enhance disease management and improve yields in sugarcane farming.

Yik-Tian Ting and Kah-Yoong Chan conducted experimental research on integrated IoT solution tailored for fruit and vegetable farms [20]. The aim of the work is to improve productivity and tackle challenges encountered by the local farming industry. The solution integrates sensor-based monitoring and control mechanisms to effectively manage various aspects of crop cultivation, facilitating seamless data collection. Moreover, the study evaluates the efficacy of utilizing long-range physical communication technique (LoRa) for transmitting sensor data through IoT gateways. Specifically, it investigates how factors such as LoRa frequency, distance, environment, and weather conditions influence the performance of LoRa-enabled IoT solutions for smart farms. Embracing simplified IoT technologies enabled by LoRa can help the agriculture sector achieve enhanced productivity, reduced operational costs, and efficient methods for ensuring food security.

A study introduced by S Mishra et al. about an intelligent animal monitoring service designed for zoos and aquariums, employing RFID, GPS, and sensor technologies [21]. Sensor nodes are utilized to measure animal body temperatures, GPS is employed for animal tracking, and RFID is used for animal identification. A prototype of the proposed tracking service is developed and tested within a service scenario.

A project in India on automatic hydroponic system is developed to decrease farming and land costs through an IoT platform connected to an Android mobile app [22]. To monitor these systems automatically, the researchers have installed pH, temperature, EC, and water flow sensors on the water tank. The water flow in each pipe is monitored using ultrasonic sensors, and the required time is recorded. However, the results do not show any significant outcomes from the implementation, except for a minor improvement in plant yields, as indicated by a comparison.

Adoption of greenhouse farming technology in agriculture has increased the net crop yields as well as return in many countries, for example, the research show that implementing this technology helped small-scale vegetable farmers increase their crop yields, net farm earnings, and per capita spending by 21%, 15%, and 13%, respectively [23]. Nahla Sadek et al. introduced a novel intelligent hydroponic and aeroponic greenhouse system utilizing IoT technology [24]. Within the indoor environment, a collection of IoT sensors is installed to monitor different factors including temperature, humidity, luminous intensity, total dissolved solids, and control a pesticide spraying tank. Findings demonstrate that optimizing energy for cultivating Batavia lettuce can lead to approximately 80% reduction in water usage. Bouali Et-taibi et al. have developed an irrigation system that uses cloud technology to link multiple small smart farms, centralizing crucial agricultural data [25]. The system was implemented using cloud based SIS - linking distributed farms using big data. All the sensors data were saved in control center database using WSN technology.

A recent study by N Yadav et al. is structured around several key modules, including data acquisition, soil moisture forecasting, smart irrigation scheduling, and an energy management scheme [26]. The primary focus is on the electrical component, which outlines an energy management strategy for a PV-battery-based grid-connected system used to operate the irrigation system’s valves and water pump. A study in California examines the performance of the Acclima smart SMS-based irrigation controller in autonomously scheduling deficit irrigation for hybrid bermudagrass using recycled water [27]. Observations from researchers indicate that maintaining soil moisture levels between field capacity and 75% of field capacity yields acceptable visual quality for hybrid bermudagrass.

Some other research has focused on water management without incorporating smart technologies. These studies include efforts such as quantifying water balances in maize fields under current irrigation schedules to enhance them using the HYDRUS-1D model [28], developing scenarios and policies to boost productivity in Pakistan [29], assessing the water quality of the Birira River and evaluating the compatibility and usability of the Biological Monitoring Working Party (BMWP) and the Tanzania river scoring system [30], and helping fishery-dependent communities on the southeastern coast of Bangladesh build resilience against environmental stresses [31].

Taken together, these studies support the notion that the inefficient use of water resources and the lack of automated irrigation systems pose challenges to indoor plant cultivation. Current irrigation systems lack optimization and automation, leading to water wastage and inefficient plant care. These studies clearly indicate that, there is a need to develop an optimized and automated irrigation system that utilizes harvested rainwater and standalone system for indoor plant cultivation. What is also not yet clear is the impact on utilizing a PV-powered system for this application. Therefore, the objectives of this research are:

- To model an optimize a PV-powered irrigation system from harvested rainwater for indoor plant application,
- To implement the automatic system using the sensing technology with an SMS-triggered function and analyze its performance of the plant irrigation.

Choosing GSM technology over other communication modules like LoRa, WiFi, or Bluetooth for smart irrigation can depend on several factors specific to the requirements of the system. Here is why GSM is preferred despite other options being more affordable: GSM technology has global availability, reliable connectivity over long distances, and established infrastructure, which makes it ideal for smart irrigation system requiring widespread network access. GSM uses existing cellular networks to enable seamless operation without additional infrastructure. Unlike LoRa, which is highly efficient for low-power and long-range IoT use but limited by region-specific coverage, GSM provides consistent performance across diverse locations without the need for additional gateways. Compared to WiFi and Bluetooth, which are better suited for short-range applications and require local access points, GSM ensures broader reach and reliable cloud connectivity, critical for real-time data transmission [33–35].

The project contributes significantly by integrating multiple sustainable technologies into a cohesive system.

- The combination of PV technology, rainwater harvest for water conservation, and GSM technology for remote monitoring creates a holistic and environmentally SIS. This integration not only reduces the dependence on conventional energy sources but also promotes water efficiency, addressing critical aspects of sustainability.
- Another contribution lies in the implementation of algorithms within the SIS. The system utilizes real-time data from sensors, including soil moisture to determine irrigation schedules. This optimization ensures that plants receive the appropriate amount of water, minimizing water wastage and promoting efficient water use include the rainwater harvesting tank that as the watering plants supply based on system modelling for water use.
- The project implementation by conducting the performance analysis of the entire system. This includes detailed testing of the PV-powered SIS, the calibration testing of soil, the integration of rainwater harvesting tank, and the enhancement with GSM technology. The analysis covers aspects such as hardware results, solar PV performance, battery discharging and charging analysis under different weather conditions. These thorough assessments provide valuable insights into the system’s efficiency, allowing for potential improvements and ensuring practical viability in real-world applications.

## System modeling

### Propose schematic diagram

**Fig 1:**
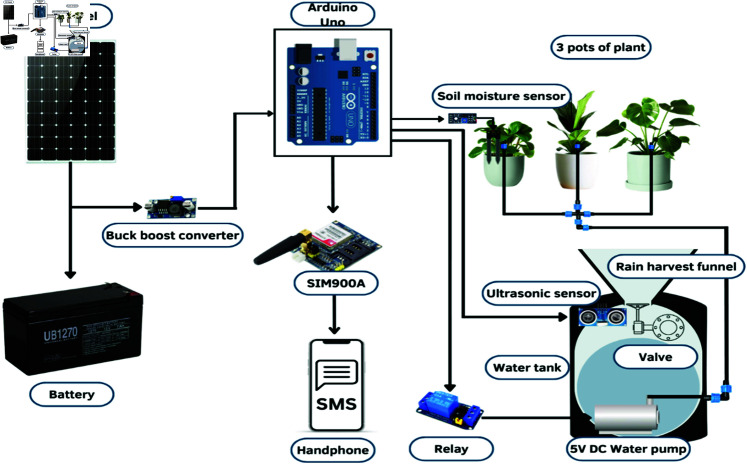
Schematic architecture of the SIS system. Soil moisture sensor is used to control the water pump. Ultrasonic sensor senses the amount of water in tank and notify users using GSM technology. Full system is powered by Solar PV, ensuring energy sustainability. Reprinted under a CC BY license, with permission from Penerbit UTHM, original copyright 2024.

Several criteria have been determined to design the effective water distribution in SIS. The criteria are rainwater harvesting systems, determine the water requirements for plants based on soil type, climate, and evapotranspiration rates, and size of the area that should be watered. Fig 1 shows the schematic design of SIS which consist of PV powered battery, moisture and ultrasonic sensors, GSM module, and sprinkler system for watering plants. Arduino UNO acts as a control hub in this system. The water pump is installed inside the tank connected with Arduino using a DC relay.

### Calibration and testing of soil moisture sensor

**Fig 2:**
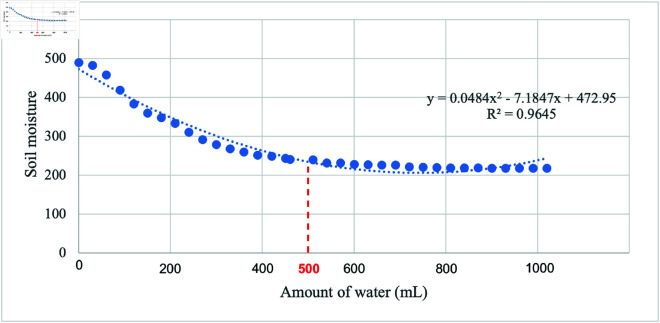
Calibration testing results between the amount of water (mL) and soil moisture. The higher serial number value from the sensor corresponds to lower moisture levels, indicating an inverse proportional relationship. The relation is best fitted (*R*^2^=0.9645) with a polynomial with degree of 2

The calibration testing for soil moisture sensor data against amount of water was conducted using 1000 g of garden soil. Fig 2 shows that the sensor reading is found to be 490 at the extremely dry level of soil, which is considered the maximum threshold of dryness. Then 30 mL of water is added gradually and observed the sensor reading. It was found that moisture saturated level was achieved when the total amount of water was 500 mL (0.5 L). At this state, the sensor reading was found to be 218. These values were considered to classify the dryness of soil and threshold for operating the water pump, explained in algorithm section.

### Modeling for rainwater harvesting tank

**Fig 3:**
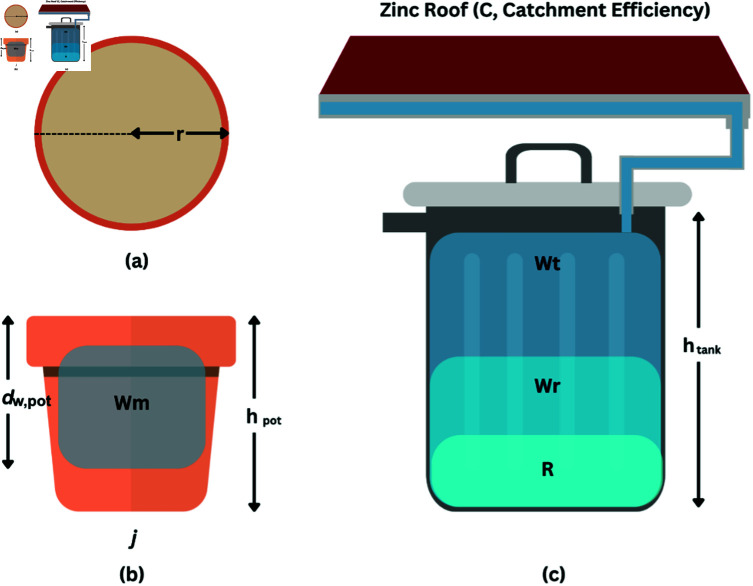
Modelling of rainwater harvesting tank- system concept and detailed layout (a) upper view of pot; (b) pot; (c) rainwater harvesting tank connect with rooftop. *W_m_*>= amount of water required for the soil in L, *r* = average radius of the upper part of pot in cm, *d_w,pot_* = soil height of the pot in cm, *h_pot_* = height of the pot in cm, *W_t_* = amount of water collected from tank in L, *W_r_* = remaining water in the rainwater harvesting tank in L, *j* denotes the number of pots, *i.e.* 1, 2, 3,… j, *C* = average catchment efficiency in %, *R* = volume of rainfall catchment in L

We model the size the of the rainwater harvesting tank to ensure that adequate amount of water is available all the time. In the modeling, we consider plant pot size, water holding capacity by soil, and amount of water required for each plant. Fig 3 illustrates the system modeling and its respective parameters.

We assume that soil volume in the pot is *V_soil_* (in L) and can be obtained from .


$$\begin{array}{ccc} V_{soil}=\pi r^{2}d_{w,pot} & & \end{array}$$ (1)

Here, *r* and *d_w,pot_* refer radius and soil height of the pot in cm, respectively.

To determine the amount of water required for each pot, the water holding capacity (*k* = 50%) is obtained from the average of peat moss soil (70%) and the garden soil (30%) [36]. shows amount of water in L required for the soil in a pot (*W_m_*) per day [37].


$$\begin{array}{ccc} W_{m}=kV_{soil} & & \end{array}$$ (2)

Now, considering *j* as a number of pots, can determine the total water required per day (*W_m_pot__*).


$$\begin{array}{ccc} W_{m_{pot}}=\overset{j}{\underset{1}{\sum }}W_{m_{j}} & & \end{array}$$ (3)

Next, we obtain the volume of rainfall catchment (*R*) per day in .


$$\begin{array}{ccc} R=AreaCatchment\times Rainfallintensity & & \end{array}$$ (4)

The average value of moderate type of *RainfallIntensity* in Malaysia within one hour is 20.5 mm or 0.0205 m. The area of catchment for rainwater harvesting is 0.4 m^2^ [38]. Therefore, *R* can be obtained as 0.0082 m^3^ (8.2 L) from . This value is based on assumption that there is at least one hour constant rainfall per day.

Since, the water for the plants will be supplied from the rainwater, it is essential to consider rainfall and the size of the harvesting tank. For this, the average catchment efficiency of the harvesting system, *C* is obtained 82.5% from Ref. [39]. Finally, the amount of water collected per day in the rainwater harvesting tank, *W_t_* can be shown in . This yields *W_t_* = 6.765 L per day.


$$\begin{array}{ccc} W_{t}=CR & & \end{array}$$ (5)

Since *R* can be varied and depend on rainfall of a day, the total amount of water in the rainwater harvesting tank per week can be denoted as *W_t_week__*. Therefore, the total number of days (*t_day_*) required to finish water from the rainwater harvesting tank can be shown in .


$$\begin{array}{ccc} t_{day}=\frac{W_{t_{week}}}{W_{m_{pot}}} & & \end{array}$$ (6)

is valid if,

- initially the rainwater harvesting tank is filled by rainwater for 7 days,
- *t_day_* can be varied subject to rainfall of the day,
- environmental conditions are neglected due to tropical region,
- size of the pots are same.

The remaining water in the rainwater harvesting tank (*W_r_*) after a week can be written as in .


$$\begin{array}{ccc} W_{r}=W_{t_{week}}-7\times W_{m_{pot}} & & \end{array}$$ (7)

In this modelling, we have determined the appropriate size of the rainwater harvesting tank concerning that the collected rainwater is sufficient for the indoor plants. To validate the model, we consider 3 pots of plants (*j* = 3), where the radius and soil height of each pot are *r* = 6 cm, and *d_w,pot_* = 9 cm, respectively. This yields soil volume for each pot is *V_soil_*=1017.68 cm^3^ or 1.01 L (). Considering *k* = 0 . 5, water required for the soil of a pot can be calculated, *W_m_*=0.51 L (). Refer to the previous section (calibration and testing of the moisture sensor), this water requirement matches the achieved saturation level of 0.5 L (Fig 2). For the 3 pots of plants, the total amount of water required per day is *W_m_pot__*=1.53 L ().

As mentioned earlier, the constant rainfall every day (that is *R* = 0 . 0082 m^3^ or 8.2 L) for 7 days. Since, the average catchment efficiency *C* = 0 . 825, the amount of water collected in the rainwater harvesting tank can be calculated, *W_t_*=6.765 L per day () and *W_t_week__*=47.36 L per week. This shows that the capacity of the rainwater harvesting tank does not need to exceed 47 L. This model also determines the optimal size of the rainwater harvesting tank. Based on this outcome, the capacity of tank is chosen to be, *W_t_week__* = 36 L, which means any additional rainwater will be drained off. Initially the tank can be empty, and it will require approximately 5.2 h of rainfall to make it full. After the tank is full and there is no rain for a week, the rainwater harvesting tank can support up to 24 days to watering the 3 pots of plants, by referring to . On the other hand, if tank is full and no rain for a week, the remaining water in the tank can be calculated, *W_r_*=36−(7∗1.53)=25.31 L. The step-by-step calculation for model validation is provided in Appendix A1.

### Rainwater harvesting system design

**Fig 4:**
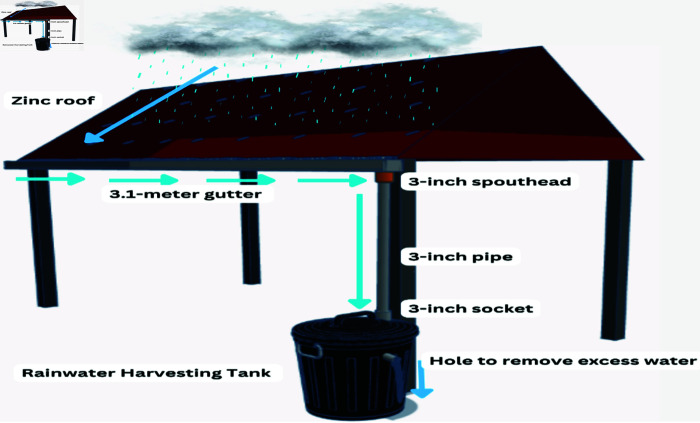
Design of rainwater harvesting system. A 3.1 m long gutter was installed at the edge of inclined roof to harvest rainwater, which then flow through a 3 inch pipe to the rainwater harvesting tank.

The rainwater harvesting system is designed to collect rainwater flowing from a zinc roof and then channeling it into the rainwater harvesting tank. The flow of water and equipment used as shown in Fig 4. We install a 3.1 *m* of gutter at the edge of the roof, which collects the water down through a 3-inch diameter pipe to the tank. A hole is created at the upper part of the tank to allow the excess water to drain out.

### Water pump modeling

The capacity of the water pump is modeled based on the system requirement shown in Fig 1. Refer to the datahseet of the pump, the water flow rate (*Q* ) 6*L* ∕ *min* or 0.1 L/s. This parameter is used to calculate the velocity of water from the micro drip pipe, based on the assumption of *W_m,pot_*.

Then the velocity of water (*v*) while sprinkling from the outer of the micro drip irrigation pipe can be written in .


$$\begin{array}{ccc} v=\frac{Q}{\frac{\pi }{4}d^{2}} & & \end{array}$$ (8)

If the diameter (*d*) of the micro drip irrigation pipe is 0.004 m, *v* can be calculated as 7.96 m/s. Referring to Fig 1 in the SIS system, we assume two water elevation locations: water pump and the irrigation pipe at plant pot, where the pressures at these locations are denoted as *P*_1_*andP*_2_, respectively. Since the atmospheric pressure at all the points of the SIS is same, the pump head (*E_p_*) can be calculated using [40].


$$\begin{array}{ccc} \frac{P_{1}}{\gamma }+\frac{v_{1}^{2}}{2g}+h_{1}+E_{p}=\frac{P_{2}}{\gamma }+\frac{v^{2}}{2g}+h_{2}+H_{lf} & & \end{array}$$ (9)

Since *P*_1_=*P*_2_=*P_atm_*, both $\frac{P_{1}}{\gamma }$ and$\frac{P_{2}}{\gamma }$ can be eliminated from both sides. *v*_1_ refers the water velocity at the rainwater harvesting tank. At the tank, the water does not move and it remains in static. Therefore, the initial velocity of the water, *v*_1_=0 m/s. The above equation can be written as [40].


$$\begin{array}{ccc} E_{p}=\frac{v^{2}}{2g}+(h_{2}-h_{1})+H_{lf} & & \end{array}$$ (10)

*h*_1_ and *h*_2_ are the height of the rainwater harvesting tank and the height between pump and plant pot. *H_lf_* is head loss reflection which can be defined in .


$$\begin{array}{ccc} H_{lf}=\frac{L\times f}{d}\times \frac{v^{2}}{2g} & & \end{array}$$ (11)

Here, *L* and *d* are the length and diameter of the micro drip irrigation pipe and its values are 1 m and 0.004 m, respectively. The constant, *f* refers friction of the pipe = 0.002. This shows *H_lf_* = 1.615. Since *h*_1_=0.5 m and *h*_2_= 0.2m, *E_p_* can be obtained as 4.55 m. Next, the capacity of the pump (*P_pump_*) can be calculated using , where, the pump efficiency, *η_pump_*=0.7.


$$\begin{array}{ccc} P_{pump}=\frac{\rho \times g\times Q\times E_{p}}{\eta_{pump}} & & \end{array}$$ (12)

This gives *P_pump_*=6.38 W, which will be sufficient size of the water pump for this system.

**Table 2:** Load analysis of all operating components for irrigation system.

Load	Power (W)	Duration (h)	Energy (Wh)
Arduino Uno R3	0.75	24	18
Capacitor soil moisture V2	0.025	24	0.6
Ultrasonic Sensor	0.15	24	3.6
SIM800L GSM	6.6	0.0003	0.009
DC Water Pump	6	0.083	0.5
Daily Consumption (*E_d_*)			22.71

### Solar PV sizing

The PV sizing is determined based on the load analysis, depicted in Table 2. The loads taken into accounts are Arduino Uno R3, moisture and ultrasonic sensors, GSM module, and water pump. This yields daily consumption (*W_d_*) is about 19.07 Wh. The sizing of solar PV (*P_PV_*) can be calculated as . Arduino, the soil moisture, and ultrasonic sensors are operating 24 h, where the GSM module does not require more than 5s to send a message. The DC pump can be operated for a maximum of 5 min (0.0833 h) when it is triggered ‘ON’ by the relay.


$$\begin{array}{ccc} P_{PV}=\frac{E_{d}}{S_{peak}}\times 1.5 & & \end{array}$$ (13)

or $\frac{22.7091Wh}{4.69h}\times 1.5$ or 7.26 W. In Malaysia, the sun hour (*S_peak_*) is found to be 4.69 h, obtained from our previous research [41]. Moreover, an additional of 50% of capacity is considered for PV sizing due to the changes in solar intensity, shading and heat generated within the PV. This shows a minimum of 7.26 W of PV is required. To determine the number of panels (*N_PV_*), a model equation presented in is considered.


$$\begin{array}{ccc} N_{PV}=\frac{P_{pv}}{P_{max}} & & \end{array}$$ (14)

or $\frac{7.26W}{30W}$ or 0.203 or 1 panel. In this project, we considered a 30 W of one monocrystalline PV panel for better performance. The specification of it is given in Table 3.

**Table 3:** Specification of 30 W monocrystalline solar PV at STC.

PV specification	
Max power, *P_max_*	30 W
Maximum power voltage, *V_mp_*	17.50 V
Maximum power current, *I_mp_*	1.72 A
Open circuit voltage, *V_oc_*	21.24 V
Short circuit current, *I_sc_*	1.88 A
Irradiance	1000 Wm^2^

### Battery sizing

The lead acid battery is selected because of its widely usage, durability, and economic benefits. To determine battery bank capacity for the project, the calculation is shown in .


$$\begin{array}{ccc} B=\frac{E_{d}}{DoD\times \eta_{batt}\times System\;Voltage} & & \end{array}$$ (15)

The depth of discharge (DoD) of the battery is found to be 50%, where the efficiency (*η_batt_*) and the system voltage are 60% and 12 V, respectively. This shows battery capacity ( *B* ) should be a minimum of $\frac{22.7091Wh}{50\%\times 0.60\times 12V}$ or 6.3081 Ah for this system.

### Maximum Power Point Tracker (MPPT)

To protect the battery from over charging by PV and draining out by the load, an MPPT is considered. The sizing of it is calculated based on the . Since one PV panel is used, number of string, *N_string_*=1 and an additional of 25% extra short circuit of PV (*I_sc_*) are considered. This shows the specification of the MPPT current (*I_CC_*) should be higher than 0.7 A. In this project, the rated *I_CC_* of MPPT is 7.8 A.


$$\begin{array}{ccc} I_{CC}=I_{SC}\times N_{string}\times 1.25 & & \end{array}$$ (16)

### Circuit diagram

**Fig 5:**
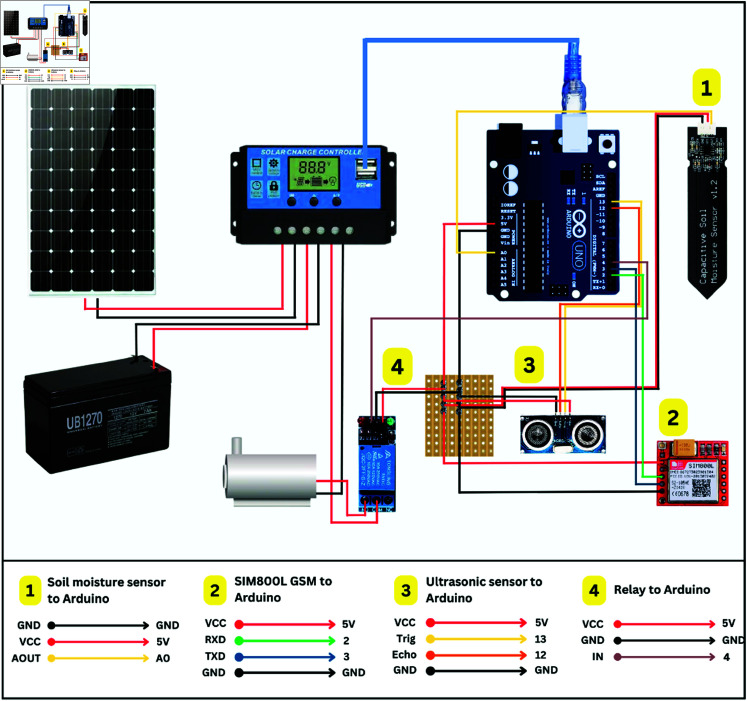
Circuit diagram of SIS. Two main components in the circuit which act as control unit- Arduino Uno and MPPT. Sensors (ultrasonic, soil moisture) and GSM module are connected to analog connection of Arduino Uno. Water pump is controlled by a relay, which is connected to digital pin. Solar PV and battery are connected to MPPT, and it ensures sufficient power to Arduino and saves battery from over charging. Reprinted under a CC BY license, with permission from Penerbit UTHM, original copyright 2024

The circuit diagram of SIS includes numerous parts and connections that make it possible for the system to work effectively, as shown in Fig 5. Since moisture sensor is analogue type, its ‘A_out_’ terminal connected to the analog input ‘A0’ of Arduino Uno. On the other hand, the SIM800L GSM module has two significant pins for data send and receive purpose, called ‘RxD’ and ‘TxD’. The two connections are established with digital pins of ‘2’ and ‘3’ respectively. The ultrasonic sensor also possesses ‘Trig’ and ‘Echo’ terminals which are connected to the digital pins ‘13’ and ‘12’ of the Arduino board. The main function of these pins is to measure distances between two objects through sound waves. The ‘IN’ terminal of relay is connected to digital pin ‘4’, enabling control over water pump based on the moisture sensor readings. Lastly, the VCC and GND of all the sensors are connected to the 5 V and ground of the board, respectively.

The MPPT acts as an optimized power controller for the SIS, connecting PV, load, and battery. On the load side, the negative (-ve) terminal of it links to the negative terminal of the water pump, and its positive (+ve) terminal connects to the ‘COM’ of the relay. The ‘NO’ terminal of the relay is then connected to the positive side of the pump.

**Fig 6:**
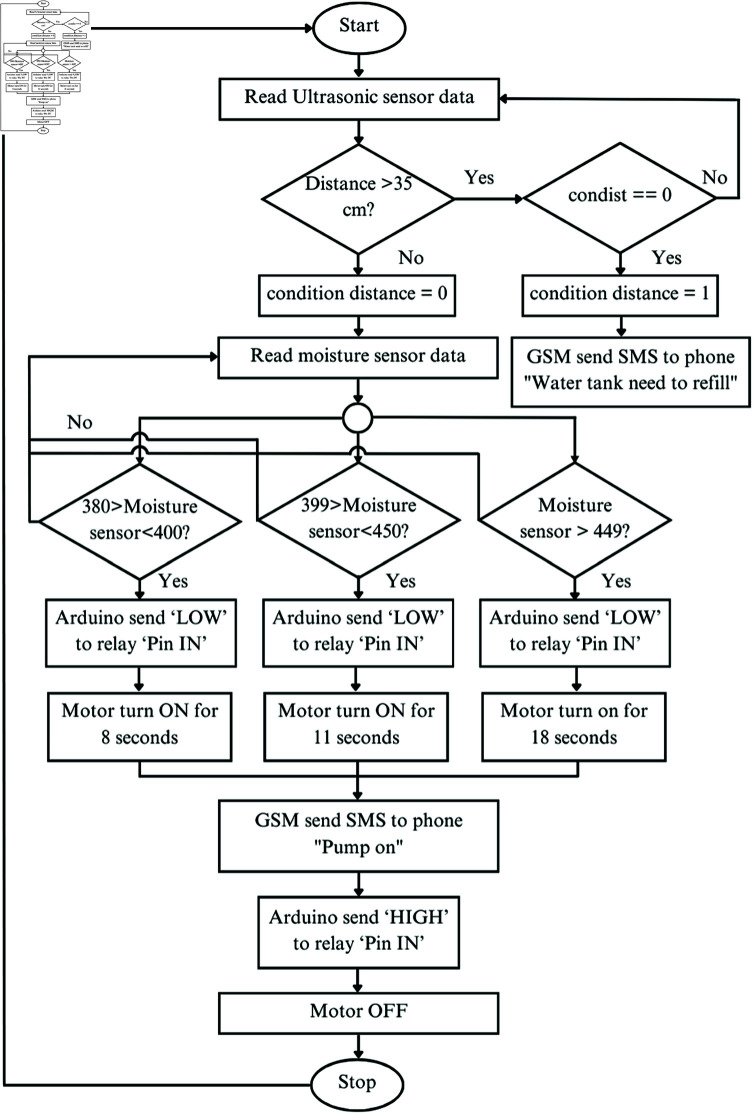
Flowchart of system algorithm. It shows smart irrigation management and pre-stage of C code development. Initially, the ultrasonic sensor measures the water depth in the container and sends an SMS to the user if the water level is insufficient. If the water level is adequate, the moisture sensor assesses the soil condition, and the water pump is activated. The user is also notified via SMS about the pump’s status.

We have developed an algorithm to ensure efficient irrigation management and provide timely user notifications. The algorithm is explained through flow chart, presented in Fig 6, which are then written in C code for Arduino microcontroller. The SIS starts by reading the ultrasonic sensor to determine the water level in the tank. If the sensor reading is ‘Height <35’, it indicates insufficient water, and the system sends an SMS to notify the user about the low water level. Otherwise (‘Height >35’), the Arduino reads the moisture sensor, which is classified into three conditions, such as very dry condition (>450), dry (400-449), and medium dry (350-399). Depend on the moisture condition, Arduino sends ‘LOW’ signal to the ‘IN’ terminal of relay through digital pin ‘4’. The water pump is then triggered by the ‘NO’ terminal of the relay for a specific duration of 18, 11, and 8 s for very dry, dry, and medium dry, respectively. Simultaneously, an SMS notification labeled ‘Pump On’ is sent to the user. Following this, the relay disconnects the pump upon receiving a ‘HIGH’ signal from the Arduino. In this state, the ‘NO’ and ‘COM’ connection of relay are open, effectively disconnecting the pump from the MPPT. Then the system initializes for the next cycle after delay a specific time.

### Algorithm development for arduino microcontroller

We have developed an algorithm to ensure efficient irrigation management and provide timely user notifications. The algorithm is explained through flow chart, presented in Fig 6, which are then written in C/C++ code for Arduino microcontroller. The SIS starts by reading the ultrasonic sensor to determine the water level in the tank. If the sensor reading is ‘Height <35’, it indicates insufficient water, and the system sends an SMS to notify the user about the low water level. Otherwise (‘Height >35’), the Arduino reads the moisture sensor, which is classified into three conditions, such as very dry condition (<450), dry (400-449), and medium dry (350-399). Depend on the moisture condition, Arduino sends ‘LOW’ signal to the ‘IN’ terminal of relay through digital pin ‘4’. The water pump is then triggered by the ‘NO’ terminal of the relay for a specific duration of 18, 11, and 8 s for very dry, dry, and medium dry, respectively. Simultaneously, an SMS notification labeled ‘Pump On’ is sent to the user. Following this, the relay disconnects the pump upon receiving a ‘HIGH’ signal from the Arduino. In this state, the ‘NO’ and ‘COM’ connection of relay are open, effectively disconnecting the pump from the MPPT. Then the system initializes for the next cycle after delay a specific time.

It is important to mention that the water insufficiency threshold of ultrasonic sensor is set at ‘35 cm’ based on calibration testing. Moreover, the soil moisture thresholds of 450, 400 and 350 are determined through soil testing with varying water quantities. The testing results are explained in the result section .

### Project implementation

**Fig 7:**
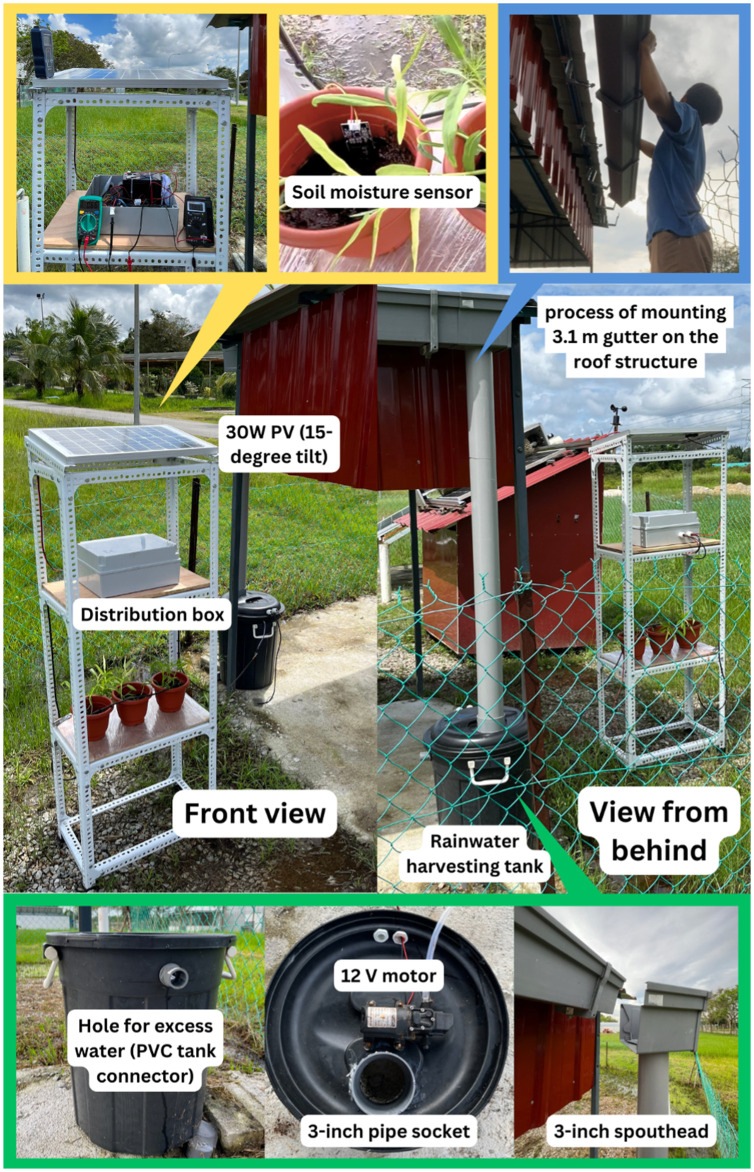
Implementation of SIS at the project location. Location coordinates [1.864860, 103.089430]. The complete system is implemented and tested over an extended period. Reprinted under a CC BY license, with permission from Penerbit UTHM, original copyright 2024.

The project has been implemented under the premises of Faculty of Electrical and Electronic Engineering, Universiti Tun Hussein Onn Malaysia. Fig 7 shows the implementation of the project which includes the rack for the plants, solar PV, distribution box containing SIS, and the rainwater harvesting tank. The research was conducted in January 2024 on a dedicated experimental site of one acre, where an indoor plant structure was established. Additionally, a rainwater harvesting system was implemented at the location. The coordinates of the site are 1.864860, 103.089430.

We placed all of the components in the distribution box neatly, such as Arduino Uno, relay, battery, MPPT, and GSM module. To mount the PV on the top rack at an angle of 15^o^, we used 3 cm × 3 cm slotted angle steel bar. The middle and third racks were allocated for placing distribution box and three pots of plant. The 3.1 m gutter was installed under the edge of the roof of the cabin. One side of the gutter is attached to a 3-inch spout head, which is then linked to the rainwater harvesting tank through a 3-inch PVC pipe. The water pump and ultrasonic sensor were installed under the lid of the tank. Water from the pump was distributed to the 3 pots using a micro drip irrigation pipe.

A systematic approach is taken to do an analysis of electrical performance of PV, battery, and overall system. To measure irradiance, a solar meter is placed next to the solar PV ensuring no shadow falls on the surface of the panel. To measure voltages, we used multimeter in parallel with the MPPT. For current measurement, the multimeter is connected in series between the PV and the MPPT. The data collection was conducted from 9:00 AM to 5:00 PM.

## Results and analysis

### Testing results of ultrasonic sensor

**Fig 8:**
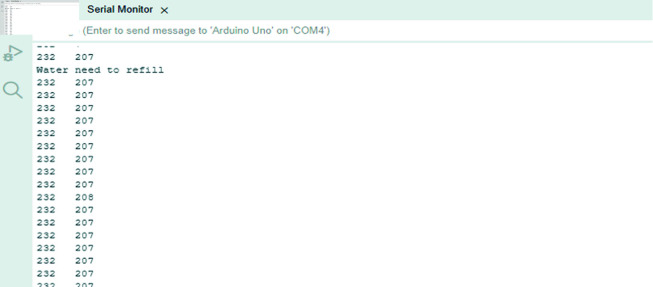
Notification message using GSM to the user when the water tank is at an insufficient level.

The ultrasonic sensor was tested by placing it under the lid of the rainwater harvesting tank, size of 36 L. Initially, the container was empty and then gradually 1.8 L of water was being added. For each time, data from the sensor was recorded. For the ultrasonic sensor testing and calibration, we observed the sensor reading was between 7-29 cm when amount of water in the task is sufficient. However, when the reading >35, the water gets sufficient. While integrating with the GSM module, the system sends warning message to the user when the water level reaches less than 7.2 L. The notification is triggered to alert the user that the water in the tank is insufficient. A *‘Water needs to be refilled’* text message is sent to the user through GSM technology, as shown in Fig 8. At this point, the water pump will not turn on until the water is refilled by the user or rainwater reaches a sufficient level.

**Fig 9:**
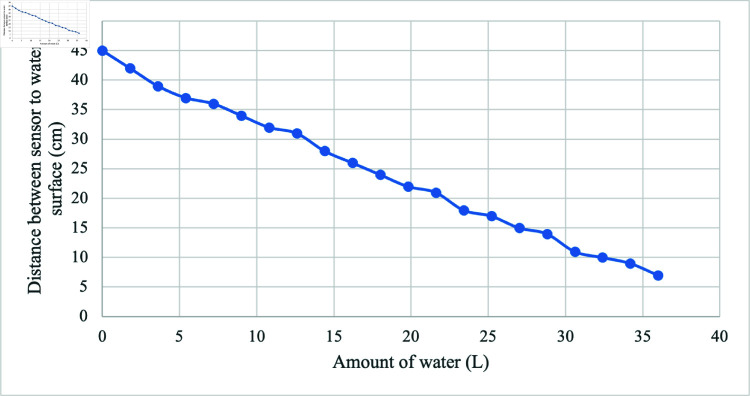
Linear but inversely proportional relationship between the amount of water (L) against distance between the sensor and the water surface (cm).

Fig 9 depicts that the distance between sensor and water surface was 36 cm at 7.2 L of water level. This value was used in the algorithm development to indicate the rainwater harvesting tank capacity. The relationship between the amount of water added and the distance between the sensor and the water surface is found to be linear and inversely proportional.

### PV performance

**Fig 10:**
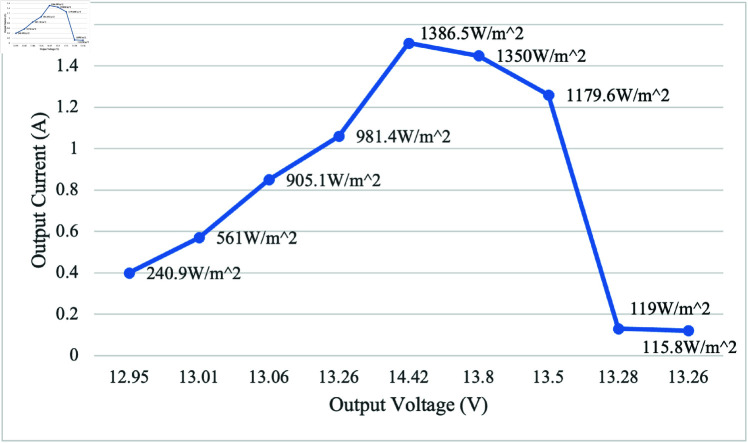
PV output current against voltage at different irradiance level. It indicates that the performance of PV is highly influenced by solar irradiance, as in general, an increase in irradiance leads to an increase in output current.

The tabulated data that shows the current and voltage including irradiance illustrates in Fig 10. It shows the performance of the PV, output current against its voltage at different irradiance on a sunny day. It is observed that output current normally increases with the irradiance. The highest and lowest current were recorded 1.51 A and 0.12 A at 1386.5 and 115.8 W/m^2^ respectively. The voltage range was found to be 12.95–14.42 V. The average power is calculated to be 11.097 W.

### Battery charging status – without PV connection

**Fig 11:**
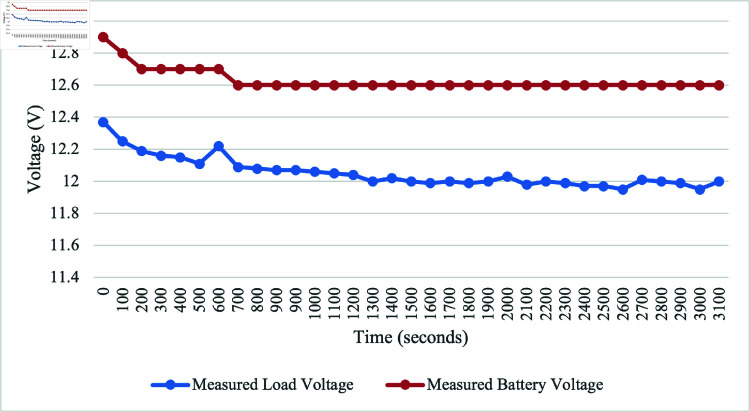
Performance of battery voltage during discharge when not connected to PV.

We have analysed the battery charging status with and without connecting to the PV panel. Without the presence of PV, the battery voltage decreases. We also observe the same scenario for load voltage (water pump). This voltage drop is due to the load drawing power from the battery. However, the measured load current changes little throughout the discharge cycle, hinting that the load is drawing its power at a constant rate from the battery. This observation is shown in Fig 11 which is indicative of the load’s power consumption continuity and stability.

**Fig 12:**
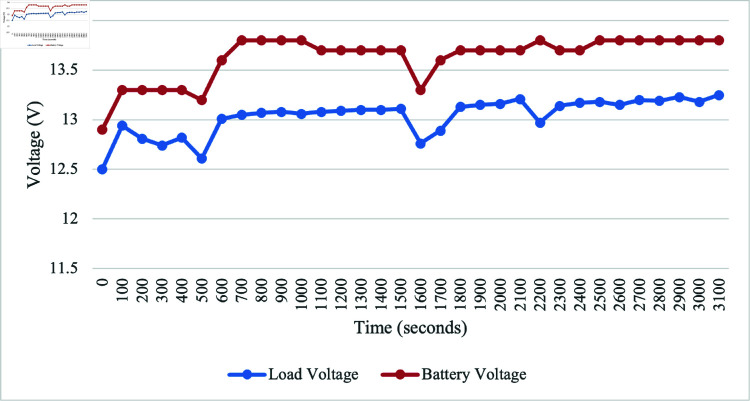
Performance of battery voltage during discharge when connected to PV.

While connecting the PV with battery and simultaneously with water pump as load, we observe both battery and load voltages are increasing over the time. Fig 12 shows the results of considering the time and the voltage for load and battery. This indicates that connecting with PV can save the battery from discharging.

### Battery charging during cloudy and moderate weather

**Fig 13:**
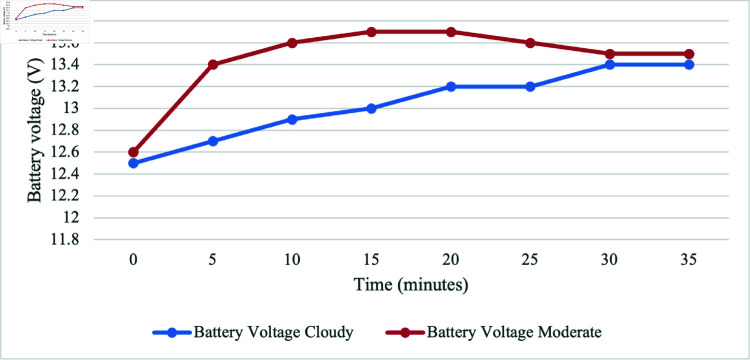
Comparison of battery voltage over time in moderate and cloudy weather.

**Fig 14:**
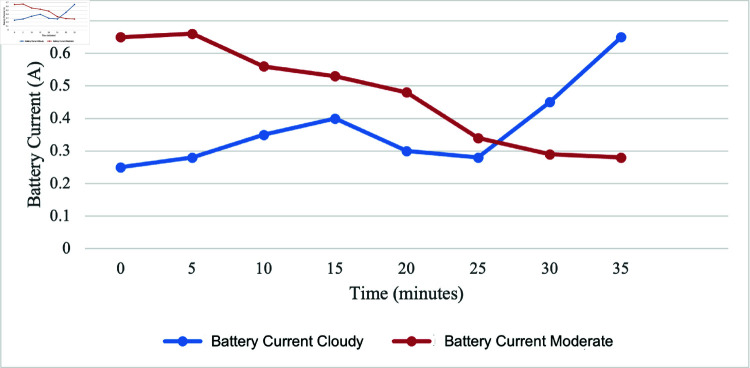
Comparison of battery current over time in moderate and cloudy weather.

In optimal conditions, output voltage of PV is directly proportional to irradiance, meaning that as sunlight gets more intense, the panel generates a higher voltage. This occurs because the increased sunlight excites electrons in the PV cells, creating a stronger electric potential across the panel. However, the scenario is different on cloudy and moderate weather. To facilitate a thorough evaluation of the battery charging status on a cloudy day, we collected data at 5-minute intervals. This organized approach allows for an in-depth analysis of how effectively the PV, in conjunction with the MPPT, charges the battery within the stipulated 35 minutes timeframe. Figs 13 and 14 show a comparison of battery charging status during the cloddy and moderate day while charging by PV. We observe that the solar PV, assisted by the MPPT, can charge the battery even in cloudy conditions since both the voltage and current of the battery was found to be a slight rise with the increase of irradiance. The PV still produces electricity, but it does so at a much slower pace than it would in sunny climate.

### Discussion on PV and battery performance

Based on the results discussed in the above sections, the highest irradiance value of 1386.5 W/m^2^ was recorded at 1:00 PM, producing the highest PV voltage and current of 14.42 V and 1.51 A, respectively. Sudden cloud cover caused irradiance to drop to 119 W/m^2^, which sharply affected the output voltage, starting at 4:00 PM. At this condition, the recorded voltage and current are 13.38 V and 0.13 A, respectively. It indicates that the irradiance plays an important role to generate higher power supply for the SIS.

For the discharging battery analysis, the initial battery voltage was 12.9 V. The maximum discharge voltage for the battery was 12.6 V after 3100 s when the discharging test was conducted without the PV connection. However, upon connecting to the PV system, the voltage increased to 13.8 V after the same 3100 s. This significant change in voltage suggests a dynamic interaction between the discharging process and the PV system. Furthermore, the next discussion focuses on battery charging under two types of days: cloudy and moderate luminance. On a cloudy day, the irradiance ranged from 250.4 to 667.8 W/m^2^, with an average of 324.24 W/m^2^. The corresponding system showed an average output voltage of 13.04 V and an average current of 0.37 A. On a moderate luminance day, the irradiance ranged from 285.5 to 928 W/m^2^, with an average of 559 W/m^2^. The system showed an average output voltage of 13.45 V and an average current of 0.47 A.

The comprehensive analysis, considering various environmental conditions such as cloudy and moderate luminance days, underscores the effectiveness of the system design. The harmonious interaction among the PV, battery, and load is evident in the consistent performance observed across these different climates. Such findings affirm the design considerations that have been incorporated into the proposed system, validating its capacity to operate seamlessly and sustainably across an environmental scenario.

### Irrigation system performance analysis

The irrigation process initiates with the soil moisture sensor serving as a crucial determinant of soil dryness, triggering the activation of the DC water pump when the soil reaches a designated dry reading. This ensures that irrigation is responsive to the specific needs of the soil, promoting water conservation and optimizing plant health. As outlined in section ‘Calibration and Testing of Soil Moisture Sensor’, the moisture level for dry soil is identified at a reading of 450, while the wet soil registers a moisture level of 200. The graph of the testing shows more to polynomial data. This testing is a baseline for understanding the soil moisture conditions that trigger the irrigation system, including to the overall efficiency and effectiveness of the irrigation process.

Next, testing was conducted to determine the level of dryness. During this testing, each of the three pots of plants received an equal and sufficient amount of water. Referring to the sections *‘Circuit Diagram’* and *‘Algorithm Development for Arduino microcontroller’* the motor was activated for 18 s to pump the 800 mL to the three pots when the soil moisture sensor reading was 408, indicating very dry condition. For dry conditions, the motor was turned on for 11 s to supply 500 mL of water when the soil moisture reading was 260. For medium dry conditions, the motor was on for 8 s to supply 300 mL of water when the soil moisture reading was 253. Based on these observations, we suggest that the soil moisture values can be set based on the type of plants. These ranges correspond to the appropriate motor activation duration and water supply volumes for effective irrigation under the specified conditions.

For the ultrasonic sensor testing, the amount of water added and the distance between ultrasonic and surface of water in the rainwater harvesting tank has been recorded. The ultrasonic sensor recorded readings between 7 and 45 cm, which is the minimum and maximum water level. As an empty tank is implied by a maximum measurement of 45 cm, this range is crucial to the system algorithm. A tank fully to capacity is represented by a minimum value of 7 cm. An interesting result is that the system detects inadequacy at a precise distance of 36 cm from the sensor, or a water level of 7.2 L, and sets off the GSM module to send the user an SMS alert informing them of the tank capacity. This functionality demonstrates the system capability for making intelligent decisions by not only tracking water levels but also analyzing them with respect to of the tank capacity. With the addition of the GSM module, the system communication capabilities are improved. The user may get real-time notifications for immediate action, which guarantees efficient operation of the rainwater harvesting system. Transitioning to rainwater harvesting testing, full capacity of the rainwater harvesting tank is 36 L, the total amount collected in a week was 52.4 L which is more than the amount of water that can fill in the tank. However, the data are based on the weather session. The highest amount of water collected is 26.1 L and the lowest is 0 due to the day was not raining. We observed that the average amount of water for three pots of the plant is 0.5 L and the water in the tank after a week is 36 L. Based on the system modeling, the number of days to finish the water in the tank is 72 days. The remaining water in the tank after a week is 32.5 L. This data prompts consideration of the system sustainability, where the observed water collection efficiency, adherence to modeled predictions, and the significant remaining capacity all contribute to the system’s viability and effectiveness in promoting sustainable water management practices.

### Carbon emission analysis

To analyze the amount of Carbon Dioxide (CO_2_) emission, the efficiency of PV is required, which is obtained from manufacturer specification (15.87%). The average power is found to be 11.097 W (referred to the section - PV performance). The sun hour in this experimental region is 4.69 h based on our previous research [41]. This yields the output energy (*E_output_*) is 52.048 Wh or 0.052 kWh per day.

A country carbon emission factor signifies the average quantity of CO_2_ emissions generated for each unit of energy produced within the country. This value represents the actual carbon emissions associated with the energy generation process. The carbon emission factor (*CO*_2,*f*_) is commonly measured in grams or kilograms of CO_2_ per kilowatt-hour (gCO_2_/kWh or kgCO_2_/kWh). The average value of *CO*_2,*f*_ is 0.788 kgCO_2_/kWh [42]. The amount of CO_2_ emissions (*CO*_2,*em*_) can be found using .


$$\begin{array}{ccc} CO_{2,em}=E_{output}\times CO_{2,f} & & \end{array}$$ (17)

This gives 0.052 kWh × 0.788 kgCO_2_/kWh or 0.041 kgCO_2_. It indicates that an amount of 0.041 kgCO_2_ can be saved from being released into the environment per day (or 14.97 kgCO_2_ per year). Assuming the total life span of the system is 10 years, 149.7 kgCO_2_ emission will be saved. This indicates a significant environmental benefit, aligning with sustainability goals and emphasizing the importance of such initiatives in mitigating climate change and promoting cleaner energy sources.

### Limitation, challenges, and economical feasibility

In this section, we explore the limitations, challenges, and economic feasibility of the study. The SIS system is composed of two main components: the electrical system (which includes the control system, solar PV, and water pump) and the rainwater harvesting system (which consists of the physical piping structure, harvesting tank, and drip irrigation to the plants). The novelty of our developed SIS with rainwater harvesting technique lies in its innovative sustainable approach, seamlessly integrating IoT and sensing technologies: PV module, a GSM module with a soil moisture sensor, an ultrasonic sensor, and a control module. The overall cost of the system is approximately RM670 (US$139.50), though the commercialized price is expected to be significantly lower. The cost calculation is shown in Appendix A2.

The distinguishing feature of the SIS is its focus on affordability and accessibility, facilitated by the use of open-source C code and the cost-effective Arduino Uno for sensors and GSM. This innovative approach addresses a significant challenge by providing an economical solution suitable for deployment across numerous small plants, particularly in indoor settings. Although smart irrigation systems for indoor applications are currently scarce, our system is designed to address this gap using advanced sensing technology. By integrating moisture and ultrasonic sensors with a unified control unit, our system collects real-time data on plant water requirements and ensures adequate water availability in the rainwater harvesting tank.

However, we have identified some system malfunction although it is not general and easily fixable. There are minor challenges of SIS maintenance in the long term, such as replacement of sensors at their faulty condition. The PV surface should be cleaned if there is any dust fall over it to ensure maximum power output. The battery is recommended to replace when its lifespan decrease to less than 60%. Since GSM module is used, it has to be ensured that sim card is active and it has available balance in order to receive message when there is insufficient water. The limitation of the study is that since we have one moisture sensor managed for multiple plants and one control unit, all the type of the plants should be similar, means that cactus and rose plants should not be placed under this SIS system as they need different level of moisture of water.

Our prototype acts as a platform to demonstrate the practicality and potential influence of smart irrigation strategies. Moving forward, we intend to broaden our experimental scope to encompass agricultural activities, enabling more comprehensive testing and refinement of our proposed SIS. Collaborating with industry partners, research organizations, and government agencies will help us address current limitations and develop a robust platform for collective research and validation. For instance, we have partnered with Aquafarm Kundasang, in collaboration with Universiti Malaysia Sabah, to install the extended version of our SIS for smart monitoring and control using IoT. Five different types of sensors will be employed to monitor water quality and assess the suitability of the aquatic environment for the fish. These sensors will then be integrated into the system to automate the control of the water pump. This collaboration will facilitate the sharing of resources, knowledge, and infrastructure, ultimately improving the scalability and efficiency of SIS initiatives.

Additionally, this approach enables the integration of data, leading to benefits such as optimized resource management, risk handling, and greater adaptability to changing conditions by utilizing real-time data from various plant types. It equips agricultural decision-makers with the ability to accurately forecast water requirements and take proactive measures to manage risks. Beyond farm-level operations, this capability extends to policymakers, agricultural services, and industry stakeholders, providing them with data-driven insights. These insights help in formulating evidence-based policies and directing investments towards broader goals like environmental conservation and sustainable development.

### Future prospects and commercialization

To address the commercialization of the system, it is important to ensure that the pricing of the devices remains reasonable. Currently, the costs of sensors, GSM modules, and embedded systems are affordable in the market, making price a minor concern when applying the system to real-world agricultural challenges. Power consumption is another crucial factor to consider during commercialization. The energy for the water pump and control system is supplied by solar PV, which will be installed on the rooftop. However, our analysis indicates that the power output from the PV system decreases during poor weather conditions compared to normal weather. Therefore, the battery system should be designed to maintain optimal performance despite varying external conditions. Rainwater harvesting is another critical component to consider for the successful commercialization of the entire system. The water piping needs to be restructured, and the size of the water pump should be calculated according to the provided mathematical model. The overall cost of the system may vary depending on the installation location.

### Conclusion

The irrigation process commences with the soil moisture sensor playing a pivotal role in determining soil dryness. When the soil reaches a specified dry reading, the DC water pump is activated, ensuring responsive irrigation tailored to the soil’s specific needs. Initially calibration testing for moisture sensor was conducted for determining the moisture levels, designated as 450 for very dry soil, 300 for dry, 253-300 for medium dry, and 200 for wet soil. These moisture values are used for algorithm development, aligning with corresponding motor activation duration and water supply volumes for effective irrigation. The sensor calibration testing provides polynomial regression, serving as a baseline for understanding soil moisture conditions triggering the irrigation system. Based on the calibration results, the motor is set to activate for different duration and water volumes, such as 18 s and 800 mL, 11 s and 500 mL, and 8 s and 300 mL for very dry, dry, and medium dry conditions, respectively. By integrating the GSM module, communication capabilities are improved, allowing users to receive real-time notifications about empty status of the tank. For the ultrasonic sensor testing, readings between 7 and 45 cm represent the minimum and maximum water levels, respectively. The system can detect inadequacy of water at a precise distance of 36 cm and triggers the GSM module to send an SMS alert about the tank’s capacity. The comprehensive analysis of PV, considering various environmental conditions such as cloudy and moderate luminance days, highlights the effectiveness of the system design. In the solar performance testing, the results reveal the highest irradiance value of 1386.5 W/m^2^ at 1:00 PM, generating a peak voltage of 14.42 V and a maximum current of 1.51 A. However, a sudden decrease occurred at 4:00 PM due to cloudy conditions, dropping irradiance to 119 W/m^2^, resulting in a decrease in voltage to 13.38 V and a current of 0.13 A. The significance of irradiance is emphasized as it plays a crucial role in generating a higher power supply for SIS. When conducting the discharging battery test, the initial voltage is found to be 12.9 V. The battery discharged to a maximum of 12.6 V after 3100 s when not connected to the PV. Once connected to the PV, the voltage increased to 13.8 V, indicating a dynamic interaction between the discharging process and PV system. Subsequent data collection involved battery charging over 2 days with varying irradiance levels: one on a cloudy day with irradiance ranging from 250.4 - 667.8 W/m^2^ and another on a moderate luminance day with irradiance ranging from 285.5 - 928 W/m^2^. On the cloudy day, the system showed an average output voltage of 13.04 V and an average current of 0.37 A, while on the moderate luminance day, the average output voltage was 13.45 V, and the average current was 0.47 A. These findings validate the design considerations incorporated into the system, affirming its capacity to operate seamlessly and sustainably across diverse environmental scenarios. Given the use of off-grid solar, the project contributes to preventing this considerable amount of CO_2_ from being released, signifying a noteworthy environmental advantage. For the CO_2_ emission analysis, it would release 0.041 kgCO_2_ into the environment daily if the system is connected to the grid. Assuming a total system lifespan of 10 years, the project would save 149.7 kgCO_2_ in carbon emissions. This aligns with sustainability objectives, underscoring the significance of such initiatives in addressing climate change and advocating for cleaner energy sources.

The research outcomes present significant benefits for the agricultural industry, particularly in the adoption of sustainable energy practices. The detailed analysis of PV performance under varying environmental conditions in Malaysia validates the effectiveness of the system design, ensuring reliable energy generation even during less favourable weather. By optimizing the PV system to function efficiently across diverse conditions, the industry can achieve consistent power supply for SIS, leading to improved water management from rainwater and crop productivity. Moreover, the ability of the PV system to dynamically interact with the battery, ensuring stable voltage levels, highlights the potential for sustained operation, reducing the need for grid dependency. The industry’s transition to such systems not only improves operational efficiency but also aligns with global benchmarks for sustainability. The reduction of nearly 150 kg of CO_2_ emissions over a decade, although seemingly modest, represents a meaningful step toward achieving broader climate goals. If scaled across large agricultural operations, the cumulative impact could be substantial, significantly reducing the industry’s carbon footprint and setting a benchmark for sustainable practices in agriculture.

## Supporting information

S1 Table Modeling of rain harvesting tank - validation of rainwater harvesting tank model.
(PNG)

S2 Table System cost calculation. (PNG)

S3 Table Rainwater harvesting data. Amount of rainwater harvested daily for one week, location: FKEE, Johor, Malaysia. The rainwater harvesting data was tested over a week. Each day at 7.00 AM, the rainwater collected in the tank was measured. After recording the measurements, the tank was drained to prepare it for the next day’s data collection.
(PNG)

## Acronyms

V_soil_: Soil volume in the pot
r: Radius of the upper part of pot
h_pot_: Height of the pot
W_m_: Amount of water required for the soil
W_t_: Amount of water collected from tank
W_t_week__: Amount of rainwater harvesting tank per week
W_r_: Remaining water in the rainwater harvesting tank
W_m_pot__: Total water required per day
d_w,pot_: Water depth in the pot
R: Volume of rainfall catchment
C: Catchment efficiency
t_day_: Total number of days required to finish water
v: Water velocity at micro drip irrigation pipe
*v*_1_: Water velocity at the rainwater harvesting tank
Q: Water flow
*P*_1_, *P*_2_: Pressures at two different locations
E_p_: Pump head
*E*_1_: Height of the rainwater harvesting tank
*E*_2_: Height between pump and plant pot
H_lf_: Head loss reflection
f: Friction of the pipe
L: Length of the micro drip irrigation pipe
d: Diameter of the micro drip irrigation pipe
P_pump_: Capacity of the pump
η_pump_: Pump efficiency
η_batt_: Pump efficiency
E_d_: Daily energy consumption
P_PV_: PV size
N_PV_: Number of PV panel
S_peak_: Sun hour
E_out_: Output energy
B: Battery bank capacity
I_cc_: MPPT current
I_sc_: Short circuit current of PV
*CO*_2,*em*_: Amount of CO_2_ emissions
*CO*_2,*f*_: CO_2_ emission factor
