# Supplementary figures and images for: Sustainable Smart Irrigation System (SIS) using solar PV with rainwater harvesting technique for indoor plants
Source: PLoS One. 2025 Mar 21;20(3):e0316911. doi: 10.1371/journal.pone.0316911 (PMC11927913; doi:10.1371/journal.pone.0316911)

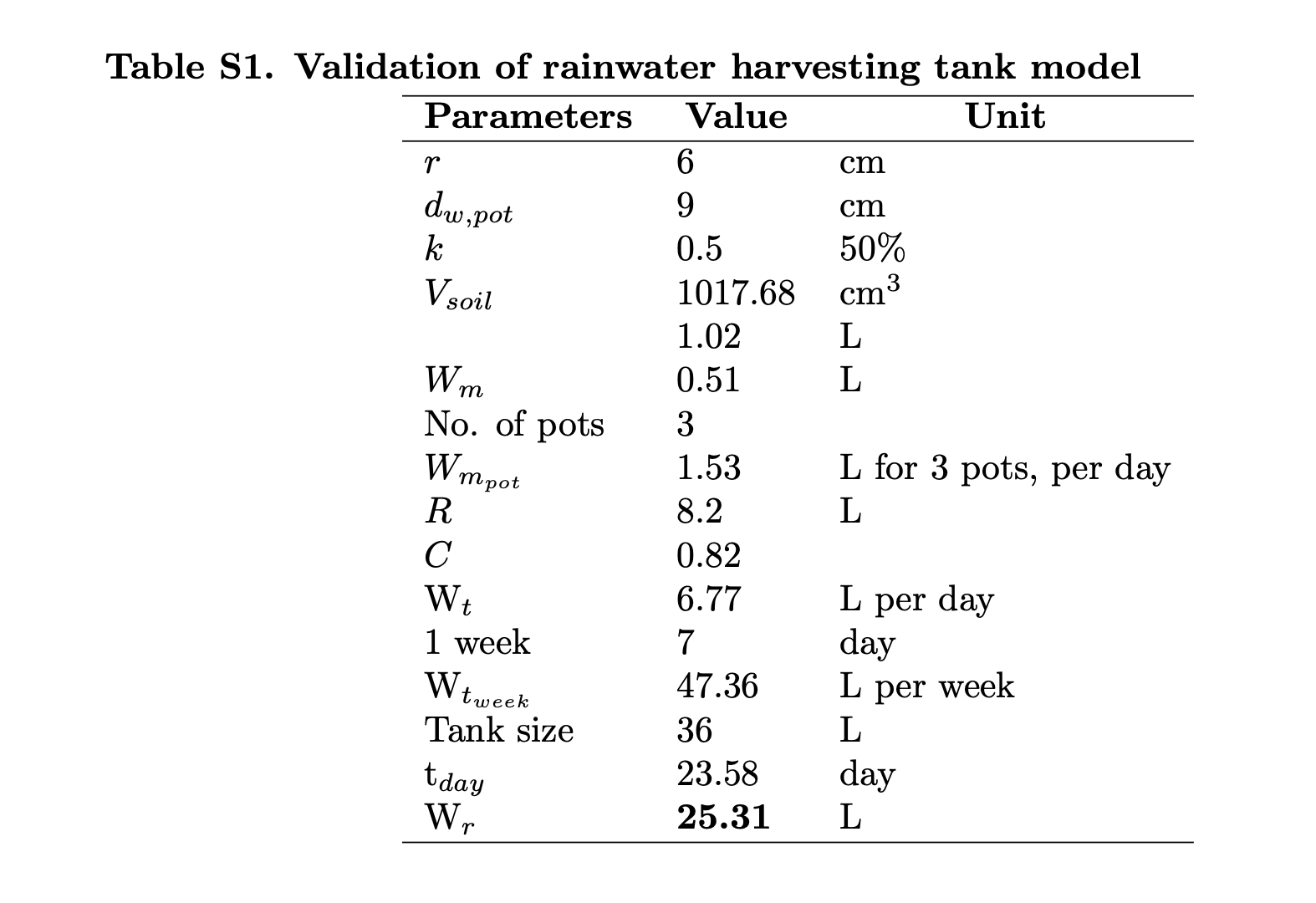

Supplement: S1 Table — (PNG) [file pone.0316911.s001.png]

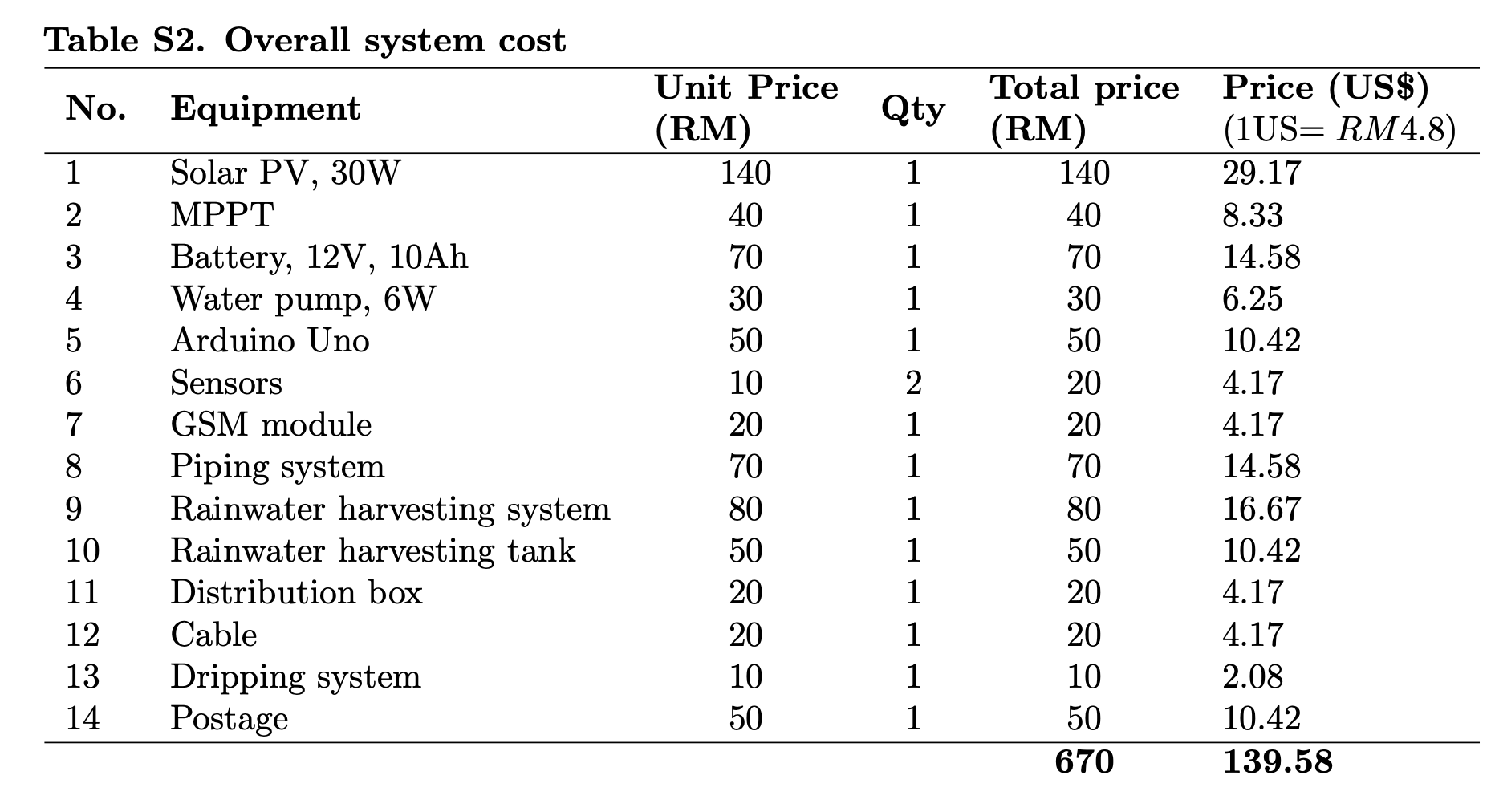

Supplement: S2 Table — (PNG) [file pone.0316911.s002.png]

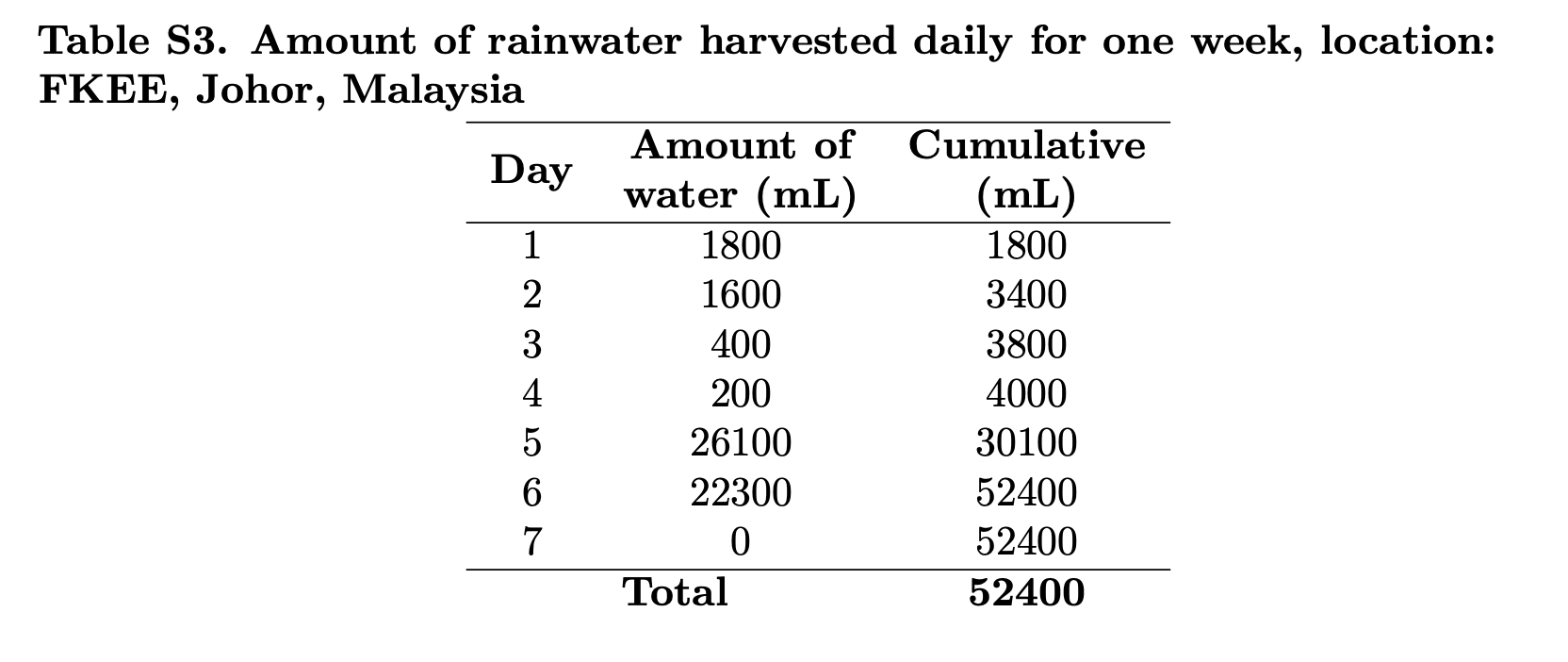

Supplement: S3 Table — Amount of rainwater harvested daily for one week, location: FKEE, Johor, Malaysia. The rainwater harvesting data was tested over a week. Each day at 7.00 AM, the rainwater collected in the tank was measured. After recording the measurements, the tank was drained to prepare it for the next day’s data collection. (PNG) [file pone.0316911.s003.png]
